# Influence of Sample Preparation Methods for Porcine Tissue: Calibration‐Free LIBS for In Vivo Applications

**DOI:** 10.1002/jbio.70327

**Published:** 2026-07-29

**Authors:** Edith Böhmer, Oana‐Maria Thoma, Maximilian J. Waldner, Florian Klämpfl, Michael Schmidt, Martin Hohmann

**Affiliations:** ^1^ Institute of Photonic Technologies (LPT) Friedrich‐Alexander‐Universität Erlangen‐Nürnberg (FAU) Erlangen Germany; ^2^ Graduate School in Advanced Optical Technologies (SAOT) Erlangen Germany; ^3^ Department of Medicinie 1 Friedrich‐Alexander‐Universität Erlangen‐Nürnberg (FAU) Erlangen Germany; ^4^ Germany Center of Immunotherapy, Deutsches Zentrum Immuntherapie (DZI) University Hospital Erlangen, Friedrich‐Alexander‐Universität Erlangen‐Nürnberg (FAU) Erlangen Germany

**Keywords:** calibration‐free laser‐induced breakdown spectroscopy, deionised water, elemental composition, laser‐induced breakdown spectroscopy, nitrogen, PBS, sample preparation

## Abstract

Calibration‐free laser‐induced breakdown spectroscopy (CF‐LIBS) has the advantage of eliminating the need for complex sample preparation. This study investigates the impact of various preparation techniques on porcine tissue. These methods include embedding the tissue in PBS or deionised water, and analysing untreated tissue or tissue that has been cut using liquid nitrogen. The results demonstrate that embedding samples does result in changes to the LIBS spectra. Minor deviations in the magnesium and sodium intensity ratios were observed in the skin. However, it should be noted that embedded samples may exhibit altered elemental signatures due to ionic exchange with the immersion medium, despite facilitating sample handling. It is important to note that the immediate measurement of untreated samples is critical, as post‐mortem changes can lead to shifts in elemental concentrations. These findings show that CF‐LIBS can be reliably performed on untreated biological samples, thus supporting its use in vivo diagnostics.

## Graphical abstract

This study examined whether the preparation of samples for CF‐LIBS influences the results of measurements taken on biological samples. Porcine skin, muscle and fat were analysed. Some samples were cut with a scalpel, some were sectioned using liquid nitrogen and some were embedded in PBS or deionised water. It was observed that the behaviour of embedded samples was similar. However, the intensity of fresh samples changes over time after the animal dies.

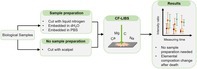

## 1 Introduction

A key advantage of laser‐induced breakdown spectroscopy (LIBS) is that, unlike many other analytical methods, it requires no sample preparation [1]. This characteristic makes LIBS a particularly fast, flexible and cost‐effective method of elemental analysis [2, 3]. As a result of these advantages, LIBS has become increasingly established as a powerful tool in a wide range of applications, including the metals industry, archaeology, geology and, in particular, medicine [4, 5, 6]. LIBS works by focusing a pulsed laser beam onto the surface of a solid, liquid or gaseous sample [1]. The high energy of the laser causes the atomic bonds to break, removing a few micrometres of material and forming a plasma [7]. As the plasma cools, the atoms, ions and molecules it contains recombine and emit element‐specific spectral lines, which are detected by a spectrometer and converted into a spectrum [8]. From this spectrum, the elemental composition of the sample can be determined [9]. The quality of the spectra produced is highly dependent on several factors, including the gate width, delay time, laser energy and the repetition rate of the laser pulses [1, 7, 10]. But also, the chemical and physical matrix effects of the sample have an influence [11]. Chemical matrix effects include differences in density and elements due to factors such as age, sex and diet [7]. Physical matrix effects include the hardness of the sample and its topography [1, 10]. These matrix effects can also change within the sample itself, leading to different laser–material interactions and subsequently influencing the LIBS result [10]. This results in lower reproducibility of LIBS compared to other methods [12].

To improve the reproducibility of the spectra, several sample preparation techniques have been developed, especially for complex biological samples [10]. Such preparation techniques include drying, embedding and freezing, depending on the type of tissue [13]. These techniques aim to minimise the matrix effects of the samples and to provide uniform ablation to achieve reproducible results [14].

In the case of biological samples in particular, which are characterised by structural heterogeneity and complex composition, it is unclear to what extent sample preparation influences the elemental composition of the sample. Since LIBS determines the elemental composition of the sample, and all embedding media are composed of elemental constituents themselves, chemical exchange between the sample and medium is unavoidable. This study, therefore, investigates how different sample preparation methods alter the elemental signature of biological tissue. It also evaluates the compromise between improved handling and reduced measurement variability.

## 2 State of the Art

**FIGURE 1:**
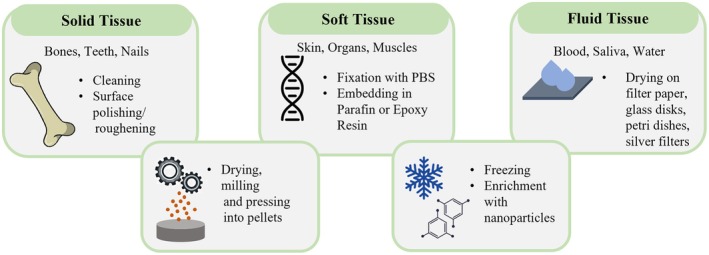
Graphical overview of the different sample preparation methods for biological samples. For solid tissue, the surface can be processed, while soft tissue can be fused or embedded. Solid and soft tissue can be processed into pellets. Liquid tissue can be dried or frozen like soft tissue. Based on [16, 17, 18, 19].

When it comes to LIBS sample preparation, it is useful to distinguish between biological and non‐biological samples. For metallic samples, preparation is generally limited to simple procedures, such as cleaning and polishing the surface, to increase reproducibility [10]. Alternatively, the surface can be roughened to improve laser–material interaction; in this case, the surface roughness is typically within the Rayleigh length. Thus, the material is always ablated uniformly with the same laser energy [10]. Due to their planar structure and, in many cases, homogeneous material distribution, metallic samples generally pose no particular challenge in terms of reproducibility of LIBS results [12]. Biological samples, on the other hand, are much more complex to handle due to their heterogeneity, irregular surface structure and poorly characterised elemental composition [6, 7]. They even produce weaker LIBS signals compared to inorganic materials [15]. Depending on their physical composition, solid tissues (teeth, bones, nails), soft tissues (organs, skin), or liquid tissues (blood, mucus, urine), different preparation techniques are used, such as cleaning, powdering, embedding, soaking or freezing; an overview of these methods is shown in Figure 1 and explained in more detail below [16, 17, 18].

### 2.1 Solid Tissue

For the analysis of hard tissue, polishing the sample surface has been shown to significantly improve the interaction between the laser beam and the material [10, 19]. To increase sample homogeneity and reduce physical and chemical matrix effects, biological materials can be dried, pulverised and then pressed into pellets [18, 20]. This reduces variability in plasma formation and improves the reproducibility of spectroscopic data [21]. However, it should be noted that changes in surface structure and heterogeneity, especially in the range of several hundred micrometres, can also cause new matrix‐related spectral deviations [21].

Homogenisation by pulverisation improves the vapourisation and atomisation of the material in the plasma, particularly when the samples are pulverised to a fine powder [10]. Janovszky et al. [22] successfully demonstrated the application of this technique by pressing pig brain into pellets to differentiate between grey and white matter using LIBS. Han et al. [23] showed that pellet samples have higher intensities than fresh tissue samples. Sample pelleting is also widely used in plant biology; for example, Jull et al. [18] investigated the influence of sodium (Na) concentration on plant growth using pelleted samples.

LIBS analysis of bone requires extensive sample preparation due to the close association with soft tissue. Tariq et al. [24] have developed a multistage cleaning protocol that includes mechanical tissue removal, boiling in water (H_2_O), acetone treatment and drying. For the study of teeth and nails, simple cleaning of the samples has proved successful. LIBS has been successfully used in vivo for the detection of caries [25]. Gaudiuso et al. [26] showed that it is possible to detect cancer from the elemental composition of nails.

### 2.2 Soft Tissue

Understanding the elemental composition of different types of soft tissue is particularly important in laser medicine and cancer diagnostics. In laser‐based surgical procedures, the unknown tissue type and incision depth pose potential risks. LIBS provides real‐time tissue differentiation and can help avoid unwanted tissue damage [27]. Numerous studies have shown that the elemental composition of different types of soft tissue differs significantly [2].

However, the inherent heterogeneity and non‐regular surface of soft tissues, as well as their potential coverage with body fluids, make LIBS analysis considerably more difficult. In vivo, biological samples are covered by a layer of blood, mucus or water [28]. These fluid films present a significant challenge for LIBS analysis, since the laser beam initially interacts with the fluid before reaching the sample surface itself. The presence of such liquids can significantly alter the matrix effects, the surface's optical and chemical properties, and thus affect the accuracy of the measurements and the ablation depth [29]. In such cases, plasma formation may initially contain elements from the liquid, leading to spectral interference and focus problems. For heterogeneous samples, grid‐shaped measurement methods or rotating sample techniques can be employed to obtain more representative results [10].

### 2.3 Fluid Tissue

Pure liquid samples are generally difficult to analyse using LIBS, as surface waves, splashes and shortened plasma duration have a negative influence on the measurement quality [30]. Physical effects such as plasma formation, shock waves, cavitation and liquid jet formation also make LIBS measurements more difficult [19, 30, 31]. This leads to lower LIBS signals for liquids compared to solids [32]. However, these influences can be reduced by using double‐pulse LIBS, in which two consecutive laser pulses are used to increase the plasma [18, 33]. Freezing liquid biological samples, such as blood or urine, into a solid aggregate state largely avoids the above‐mentioned effects [34]. Rapid freezing using liquid nitrogen (N) is particularly advantageous as it eliminates the need for complex optical adjustments and enables detection limits in the ppm range [34]. This maintains inherent homogeneity and improves the LIBS measurement [19]. Another advantage of freezing is that the samples are easier to handle and measure [30]. Jantzi et al. [10] demonstrated that frozen samples exhibit improved coupling with the laser pulse compared to liquid samples. This results in higher ablation rates, a higher plasma temperature and a higher electron density [35, 36]. In turn, this increases analytical sensitivity and reduces measurement variability [10]. In a comparative study, frozen samples exhibited enhanced signal strength, greater sensitivity and detection limits up to six times better for magnesium (Mg) quantification [35]. Freezing samples is characterised by high speed and low cost. However, it should be noted that freezing can alter the matrix effects, optical properties and geometric structure of the sample, which must be taken into account when interpreting the data.

In medical practice, tissue samples are often embedded in paraffin wax for long‐term preservation and to enable the production of thin sections (2–5 μm) for microscopic analysis [37]. Paraffin can cause volume shrinkage of 8%–20% and alter protein structure due to formalin fixation, which can directly impact LIBS results [37]. Nevertheless, kerosene embedding remains an established method due to its compatibility with histological stains (e.g., haematoxylin–eosin–safran, HE staining) [38]. This allows LIBS analysis to be performed directly on embedded tumour tissue, although the low mechanical stability of kerosene complicates laser ablation [38]. Epoxy resins offer an alternative embedding method that provides higher mechanical strength and thus improves ablation efficiency [38, 39]. In the geosciences and life sciences, epoxy resins are commonly employed to fix and stabilise delicate samples for techniques such as laser ablation inductively coupled plasma mass spectrometry (LA–ICP–MS), electron beam microanalysis (EPMA) and scanning electron microscopy (SEM) [10, 38]. Sancey et al. [40], for instance, analysed mouse kidney tissue embedded in epoxy resin. Bush [39] demonstrated that epoxy resin enables more efficient laser ablation than untreated tissue, with a curing time of around 2 days.

Short‐term fixation can also be achieved using a phosphate‐buffered saline (PBS) solution. PBS is the most commonly used buffer in immunocytochemistry and contains sodium chloride (NaCl), disodium hydrogen phosphate (Na_2_HPO_4_), potassium chloride (KCl), potassium dihydrogen phosphate (KH_2_PO_4_) and H_2_O [41, 42]. The solution ensures an isotonic environment and stable pH values. In experiments, PBS is primarily used as a washing solution for cell cultures [37]. For analysing samples from metal‐coded immunoassays, standard microparticle suspensions were prepared in PBS containing 1% bovine serum albumin [43].

However, it should be noted that LIBS is an elemental atomic spectroscopy method, and chemical equilibration between the elements occurs as soon as a sample is embedded in a solution. Consequently, the spectrum of certain elements exhibits lower or higher intensities. The presence of salts from PBS could also introduce additional spectral lines, complicating elemental analysis and quantification. In histological practice, deionised water (dH_2_O) is frequently used to prepare tissue sections, particularly when smoothing and unfolding sections in a warm water bath before mounting on slides [44, 45]. dH_2_O is often used as a diluent to reduce self‐absorption, provided it introduces no matrix effects or interfering elements, though excessive dilution may cause element loss [10]. Self‐absorption refers to the phenomenon where emitted photons from a spectral line are reabsorbed by atoms of the same element within the plasma. This results in a broadening and reduction of the line intensity, ultimately leading to an underestimation of the element's concentration in the sample [8]. Although dH_2_O is widely used in medical diagnostics, no studies have investigated its effect on the LIBS spectrum when used to embed biological samples. This research gap presents opportunities for future studies, particularly about optimising sample preparation for LIBS analyses.

One option is to apply the liquid to carrier materials, such as filter paper or glass slides, and then analyse them [17]. He et al. [46] used filter paper as an enrichment substrate to enhance the LIBS signal for the Li^+^ detection. The liquid can also be dried to enhance the signal‐to‐noise ratios [13]. Mehari et al. [2] proved that emission intensities are significantly lower with moist samples than with dried ones. Another approach to enhancing the sensitivity of liquid samples is to add nanoparticles. These lower the ablation threshold and promote material ablation and plasma excitation [17]. Melikechi et al. [43] have demonstrated that integrating nanoparticles can significantly increase LIBS emission.

### 2.4 No Sample Preparation

Although biological samples are not subjected to any special sample preparation, various influencing factors must be taken into account. It should be noted that the elemental composition can change depending on the sex, age and lifestyle of the patient, as was established by Skalny et al. [17] in the analysis of nails. Nutrition also influences the elemental composition of bones, as has been demonstrated in cattle [47]. Post‐mortem changes include rapid potassium (K) loss from internal organs within 12 h and calcium (Ca) accumulation, while the dissolution of untreated samples remains low [48]. A key question is how sample preparation affects its elemental composition and how this, in turn, influences LIBS results. It is also important to examine how this composition changes over time. These considerations formed the basis of this study. To address these issues, porcine tissue was chosen as a model system due to its anatomical and dermal similarity to human tissue [7, 49]. The study investigated how the elemental composition varies over a period of 3 days compared to untreated tissue, using different preservation methods, storage in PBS and storage in dH_2_O. To analyse the impact of the sample cutting procedure, some samples were cut untreated with a scalpel, while others were cut with a scalpel after being placed in liquid N. The aim was to minimise sample preparation as much as possible to reflect realistic, practical conditions. The criteria used to evaluate the effectiveness of each preservation method included changes in elemental signal intensities, measurability and sample handling.

## 3 Materials and Methods

A flashlamp‐pumped frequency‐doubled Q‐switched Nd:YAG laser system (Q‐smart 450, Quantel laser, LUMIBIRD, France) operating at a wavelength of 532 nm was used in this study. This system is capable of producing a maximum pulse energy of 150 mJ at the specified wavelength of 532 nm, with a pulse duration of 5 ns and a maximum repetition rate of 10 Hz. The laser beam is directed from the laser source through a polarising plate, followed by a lambda half‐wave plate and another polarising plate to adjust the laser energy. The laser beam is then split by a beam splitter. Splitting 3.5% of the laser energy, which is sent to the energy meter (PM100D, Thorlabs, USA). The remainder of the laser beam is focused onto the sample surface using mirrors and an objective (MY10X‐823, Thorlabs).

**FIGURE 2:**
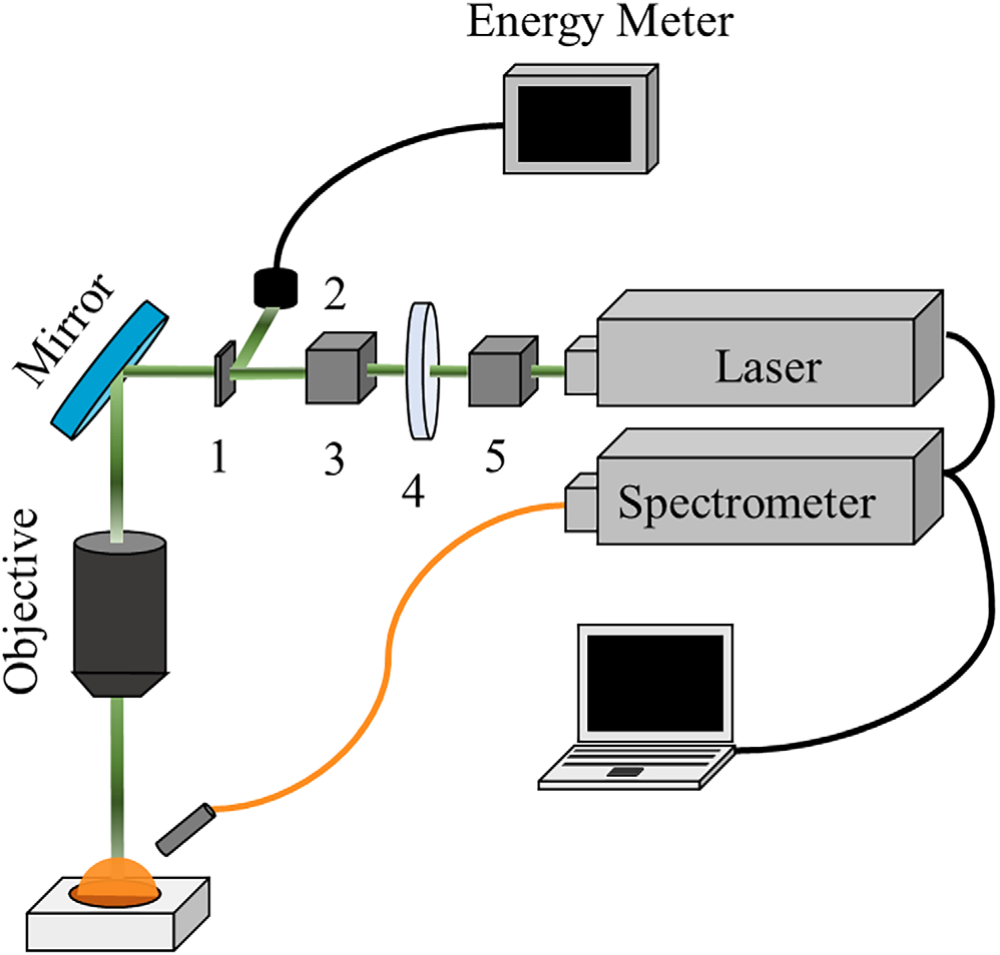
This is an illustration of the LIBS setup. Laser energy adjustment is by a measuring unit consisting of a polariser and a Lambda half‐wave plate. A beam splitter and an energy meter measure the laser energy. The laser beam is directed at the sample surface via mirrors. The high energy of the laser beam ablates material from the surface of the sample and generates plasma. As the plasma cools, it emits light which is captured by a fibre and sent to a spectrometer. The spectrometer sends the data to a PC and generates a spectrum. Also shown in [8, 9].

The laser beam was focused on the surface of the sample to minimise ionisation of the surrounding air. The emitted light was collected and directed to a spectrograph (Andor Mechelle Me5000 Echelle, Oxford Instruments, UK) without any lenses in between, with the fibre positioned approximately 5 mm from the sample to avoid capturing light from the surrounding area. The spectrograph covers a spectral range of 230–850 nm and has a spectral resolving power *λ*/Δ*λ* of 6000, corresponding to a spectral resolution of 0.1 nm at a wavelength of 600 nm. All measurements were made at ambient pressure and temperature. The experimental setup is shown in Figure 2. In this study, a laser energy of 80 mJ, a delay time of 1600 ns and a gate width of 12 μm were used; these parameters were verified in a preliminary study for CF‐LIBS in porcine samples [9].

### 3.1 Ethical Considerations

No ethical approval was required for this study. Porcine bellies were purchased as meat by‐products from a local butcher in Erlangen, Germany. No live animals were used, housed or subjected to any procedures as part of this research. Only purchased ex vivo tissue was used.

### 3.2 Biological Samples

**FIGURE 3:**
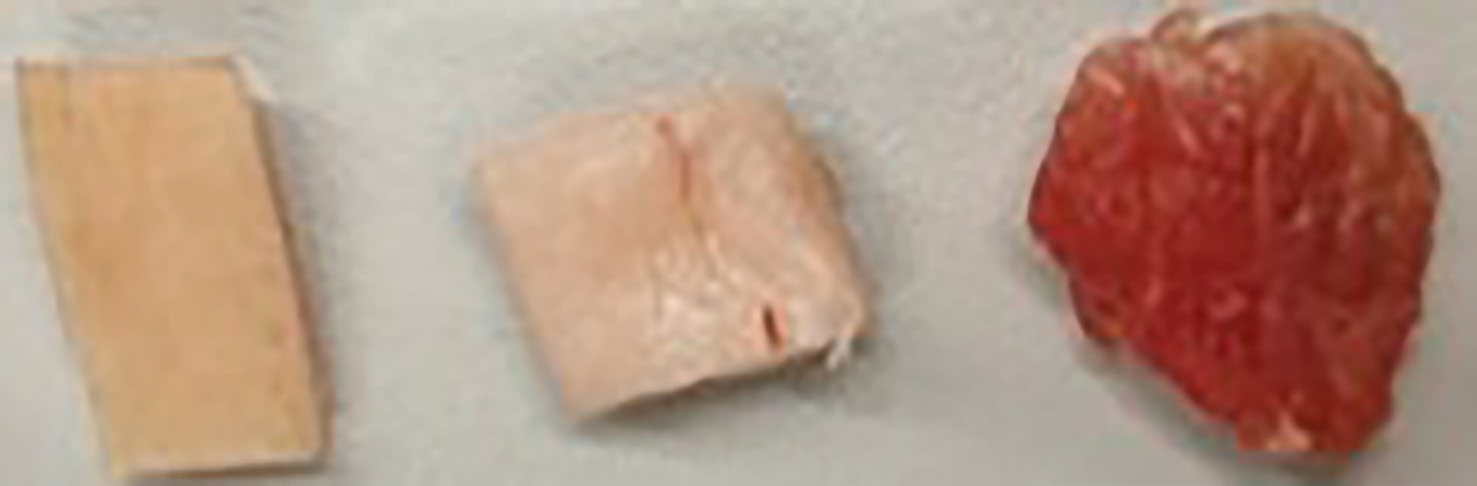
Porcine samples used. From left to right: Skin, fat, muscle.

In this study, three porcine bellies were purchased from the local butcher. The samples from three animals have been used to reduce the effects of elemental differences due to the animals' sex, age and diet. From each tissue type, porcine belly skin, fat and muscle, three samples per parameter were cut by hand with a scalpel. Figure 3 shows skin, fat and muscle from left to right. It can be clearly seen that skin has the smoothest and driest surface. To counteract any influencing factors in the chemical reactions, an attempt was made to use approximately the same sample sizes for each type of tissue. All samples were weighed for this purpose. Table 1 provides an overview of the mean weights and standard deviations of the samples. After cutting the samples, they were stored separately in tubes to avoid confusion and chemical interactions.

**TABLE 1:** The spectra used were selected from the 27 spectra generated for the untreated, PBS and dH_2_O samples, and the 9 spectra generated for the samples cut with fluid N. These are shown for fat, skin and muscle, divided into Days 1, 2 and 3. The percentage of spectra ultimately used is shown on the left.

	Weight (g)		
	Fat	Skin	Muscle
Untreated	0.653 ± 0.17	0.453 ± 0.13	1.062 ± 0.32
N	0.674 ± 0.15	0.410 ± 0.13	1.230 ± 0.25
PBS	0.729 ± 0.17	0.552 ± 0.12	1.254 ± 0.22
dH_2_O	0.546 ± 0.21	0.420 ± 0.10	0.989 ± 0.17

The untreated and liquid N samples were placed in the refrigerator at 4°C. The remaining samples were placed in 20 mL of PBS (PBS, pH 7.4, liquid, Sigma‐Aldrich, Germany) or dH_2_O, and then also refrigerated. Three spots were examined for each sample. Each spot was cleaned with a laser shot before measurement. The spot was then exposed to 10 laser shots, which were accumulated to reduce the influence of noise. After measurement, the samples were immediately cooled again to counteract any influences. Measuring a sample only takes a few minutes.

### 3.3 Element Determination

PBS consists of several elements, such as Na_2_HPO_4_, KCl, NaCl and H_2_O. These elements can lead to chemical equilibrium between biological samples and the liquid. For the evaluation of the spectra, it was decided to take only the elements magnesium (Mg) and calcium (Ca), as it is assumed that these are only found in the biological samples, not in the air, and therefore diffuse only into the liquid and not from the liquid into the tissue. Sodium (Na) was also examined because its spectral lines were clearly identifiable in the spectrum [50]. Ca was chosen because it has well recognisable intensities over a large wavelength range (350–650 nm) [50]. Mg was chosen because it has clear intensities in the lower wavelength range, and these lines cannot be confused with others [50].

Due to laser–material interaction, intensities between individual LIBS measurements fluctuate considerably, so intensity ratios were considered instead of individual intensities. In this study, the Na:C, Mg:C and Ca:C ratios were considered. Carbon (C) was taken as a reference because it is assumed not to change within tissue types or over time. In addition, C does not occur in dH_2_O or PBS. The Na:C ratio for untreated, liquid N samples and those in H_2_O is used as a reference.

Several wavelengths were selected from each spectrum according to the National Institute of Standards and Technology (NIST), generated for the four elements, as shown in Table 2 [50]. The mean values and standard deviations of the intensities were calculated for each element at each wavelength for each generated spectrum. Due to factors such as inhomogeneity and matrix effects, biological samples often have higher standard deviations compared to metals [15]. The intensity ratio was then calculated based on the total mean value for each element.

**TABLE 2:** Wavelengths *λ* of the elements used to calculate the intensity ratios from NIST. With the respective upper *E*_*k*_ and lower energy *E*_*i*_ levels and transition probabilities g⋅A, based on [50].

λ (nm)	$E_{i}$ (eV)	$E_{x}$ (eV)	g⋅A (s^−1^)	
C	290.326	8.848	13.117	3.9 × 10^6^
C	290.495	8.851	13.117	6.6 × 10^6^
C	474.257	7.946	10.560	3.0 × 10^5^
C	481.216	7.480	10.056	1.2 × 10^5^
C	530.085	8.640	10.979	5.7 × 10^5^
C	580.580	8.348	10.983	4.0 × 10^5^
Ca	428.301	1.886	4.7798	2.1 × 10^1^
Ca	442.544	1.900	4.6802	1.4 × 10^8^
Ca	518.885	2.933	5.3213	2.0 × 10^2^
Ca	526.224	2.521	4.8767	6.0 × 10^8^
Ca	558.876	2.526	4.7435	3.4 × 10^8^
Ca	612.200	1.886	3.9104	8.6 × 10^7^
Ca	616.217	1.899	3.9104	1.4 × 10^8^
Ca	649.378	2.521	4.4300	2.2 × 10^8^
Mg	273.350	2.712	7.2460	4.6 × 10^7^
Mg	281.111	5.946	10.355	9.8 × 10^5^
Mg	284.672	2.709	7.0632	3.9 × 10^7^
Mg	291.545	5.753	10.005	2.0 × 10^7^
Mg	309.689	2.717	6.7190	3.4 × 10^8^
Mg	332.992	2.709	6.4314	9.2 × 10^6^
Mg	383.230	2.712	5.9459	6.0 × 10^8^
Na	342.730	32.70	36.317	1.7 × 10^8^
Na	350.250	32.70	36.239	3.5 × 10^8^
Na	351.100	32.70	36.231	3.2 × 10^8^
Na	391.790	33.10	36.259	5.3 × 10^8^
Na	439.003	2.102	4.9257	3.9 × 10^6^
Na	466.856	2.104	4.7594	1.4 × 10^7^
Na	449.766	2.104	4.8602	8.7 × 10^6^
Na	818.326	2.102	3.6170	1.7 × 10^8^

## 4 Results

The results of the LIBS measurements are discussed below, comparing the intensity ratios of the untreated samples, the samples cut with liquid N, and the samples embedded in PBS and dH_2_O.

### 4.1 Spectra

Due to the strong fluctuations in the individual LIBS spectra during the measurements, all spectra were visually inspected for noise and baseline shifts. Each sample was measured three times, with three samples used for each preparation method and animal. This corresponds to three animals, three tissue types, three spots = 27 spectra for one preparation method and day. For the samples cut with liquid N, only one animal, three tissue types and three spots, leading to nine spectra for one preparation method and day, have been analysed. Table 3 shows how many of these spectra were ultimately used for analysis. In total, 27 × 3 × 3 = 243 spectra were generated for untreaded, dH_2_O and PBS. The preparation methods are denoted by the 3, and the measured times are 24, 48 and 72 h after the samples were purchased. This wide time range was used because the literature showed that changes in the chemical composition can still occur even after 20 days [51]. For the samples cut with liquid N, 9 × 3 × 3 = 81 spectra were generated. Each spectrum consists of 10 accumulated laser pulses to reduce the influence of noise. Good spectra were identified using a combination of visual inspection and an automated Python script for spectral quality control. Initially, the script filtered out spectra with insufficient intensity, defined as the absence of peaks exceeding 1000 a.u. This threshold was established through preliminary investigations to distinguish signal from noise. Spectra containing only a few significant spectral lines or exhibiting flat baselines, often due to misalignment or poor laser–material interaction, were excluded. Spectra with complete baseline shifts were also discarded. Following automated screening, any remaining spectra were visually reviewed to identify and exclude those with missing intensities in relevant wavelength ranges.

**TABLE 3:** The spectra used were selected from the 27 spectra generated for the untreated, PBS and dH_2_O samples, and the 9 spectra generated for the samples cut with fluid N. These are shown for fat, skin and muscle, divided into Days 1, 2 and 3. The percentage of spectra ultimately used is shown on the left.

Type	Fat			Skin			Muscle			
Days	1	2	3	1	2	3	1	2	3	(%)
Untreated	27	27	26	15	24	19	21	22	24	84.36
N	9	9	8	8	8	7	8	8	7	88.89
PBS	22	20	22	20	12	21	18	26	20	74.49
dH_2_O	12	24	23	18	18	23	16	27	25	76.54

As shown in Table 3, spectra from skin embedded in PBS produced the poorest quality results, followed by those from skin embedded in dH_2_O. By contrast, samples cut with fluid N produced the best spectra. This is because these samples have a flatter surface than the others and thus a better laser–material interaction. The flat surface also ensured that the laser remained in focus throughout, preventing baseline shifts in the spectrum and producing clear, narrow peaks. Even though the embedded samples absorbed liquid over 3 days, there was no clear tendency for the number of good spectra to decrease. In fact, the number of good spectra even increased with dH_2_O.

The observed increase in the number of good spectra over the 3 days may be explained by the samples gradually becoming more homogeneous due to elemental diffusion. This process probably led to more uniform surface conditions, improving the consistency of the LIBS measurements. Consequently, fluctuations between individual measurements decreased, and the interaction between the laser and the sample surface became more stable and predictable. It is clearly evident that skin has the lowest reproducibility compared to fat and muscle. Untreated skin samples performed the worst. Fat had the highest reproducibility and was also the easiest to handle during measurements, as Càceres et al. [30] showed. Muscle was the most challenging and also showed the greatest fluctuations in the spectrum.

**FIGURE 4:**
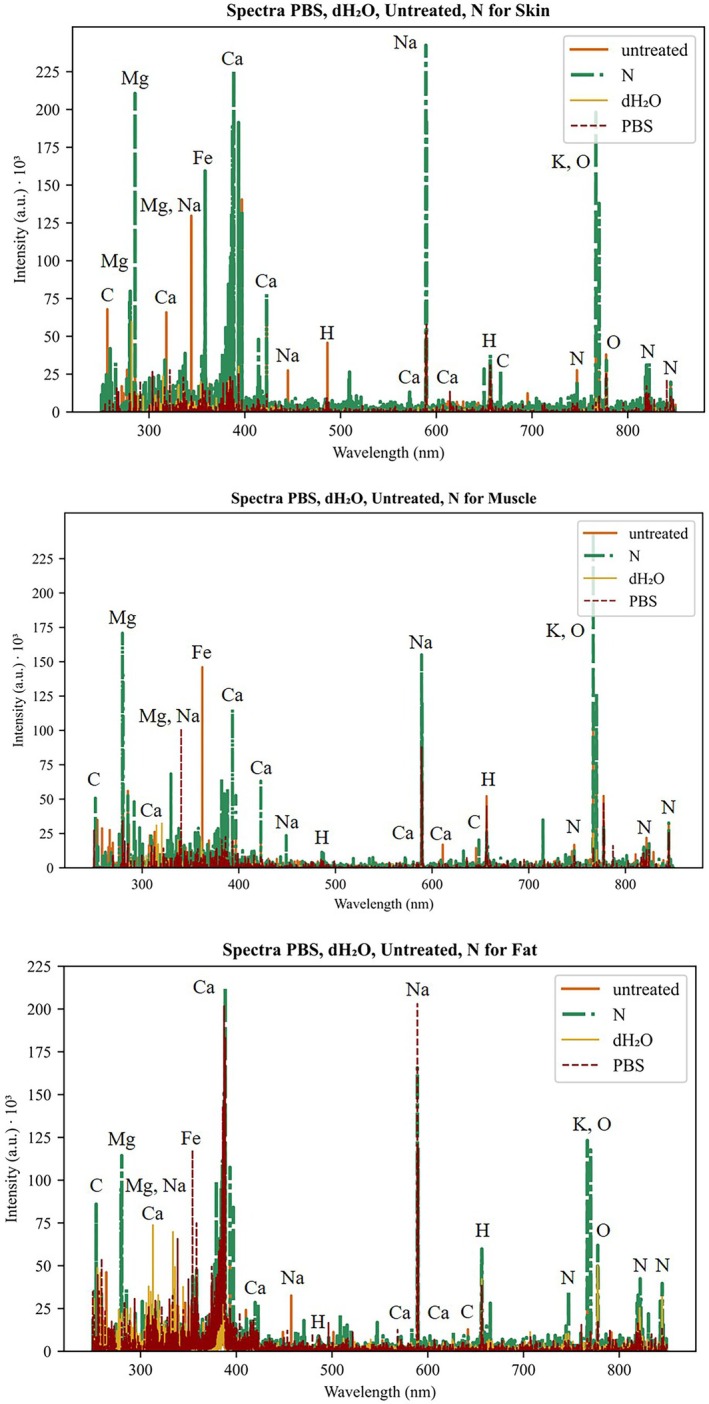
LIBS spectra of porcine skin, muscle and fat, untreated, cut in liquid N, embedded in dH_2_O and PBS on Day 1. For each tissue type, specific intensity peaks of certain elements are labelled. Intensity is on the *y*‐axis, wavelength on the *x*‐axis in nm.

Figure 4 shows the spectra of the individual tissue types compared to the sample preparation method on Day 1. The *y*‐axis shows the intensity in a.u. multiplied by 10^3^, and the *x*‐axis represents the wavelength in nm. The untreated samples are shown in red, the samples cut with liquid N in green and those placed in dH_2_O in yellow, followed by the PBS samples in red.

The first graph shows the spectra of porcine skin. Where the samples cut with liquid N have the highest intensities for Na, Ca and oxygen (O). The untreated samples also seem to have high intensities. Ca, O and nitrogen (N) are highest in the dH_2_O sample. In the untreated sample, only Mg is the highest. In skin, the Na spectral line is also the highest, followed by the hydrogen (H), O and N lines. Ca is highest in the dH_2_O sample, as is Mg. The untreated sample only has the highest spectral line at potassium (K). In the muscle samples, the Na line is also highest for the PBS samples. In the untreated samples, clearly high spectral lines are shown for Mg, Ca and K. The muscle spectrum also shows that samples cut with liquid N have the highest intensity. Clear peaks are visible for Mg, Ca, Na, K and O, whereas the untreated samples only exhibit high peaks for iron (Fe), H and O. The lowest intensities are seen for the embedded samples. For fat, high intensities are evident for Ca and Na, especially for the samples embedded in PBS and those treated with liquid N. Samples embedded in dH_2_O show high Ca intensities, whereas the untreated samples show the lowest intensities with a pronounced Na peak.

### 4.2 Ratio Na/C

**FIGURE 5:**
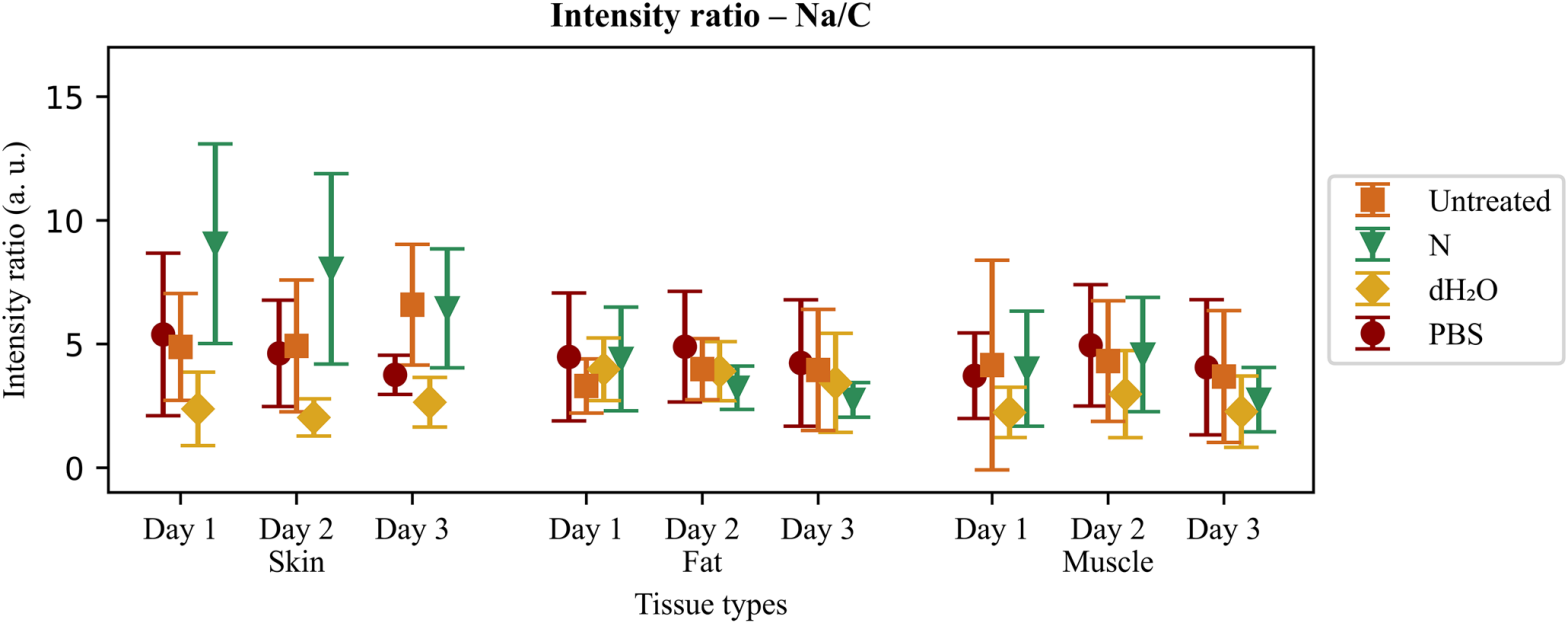
Intensity ratios of Na/C in porcine skin, fat and muscle for different sample preparation methods. The *y*‐axis shows the intensity ratio in arbitrary units; the *x*‐axis shows the tissue type and measurement time.

The following Figure 5 shows the intensity ratio of Na/C. The *y*‐axis of each graph shows the intensity ratio in a.u., while the *x*‐axis shows the measurement days and individual tissue types. Data for untreated samples is shown in orange, samples cut with liquid N are shown in green; samples embedded in dH_2_O are shown in yellow; and samples in PBS are shown in red. The first spectrum shows the Na/C intensity ratio. As can be seen, the skin liquid N samples have the highest Na values compared to the other samples. The Na content rises on the second day and then falls. The other samples behave similarly, with the PBS samples having higher Na values. For both embedded samples, the Na content decreases over 3 days, whereas it increases in the untreated samples. All tissue types behave similarly for porcine fat. Here, the untreated samples have the highest Na intensity. In all cases, there is a slight increase in intensity on the second day, which then decreases on the third day. A similar situation occurs with the muscle samples. Again, the PBS samples have the highest intensity, followed by the untreated samples. There is a slight increase on Day 2, followed by a decrease on Day 3. The increased Na intensity in the PBS samples can be explained by the presence of Na in PBS. It most likely diffuses from the PBS into the tissue.

### 4.3 Ratio Ca/C

**FIGURE 6:**
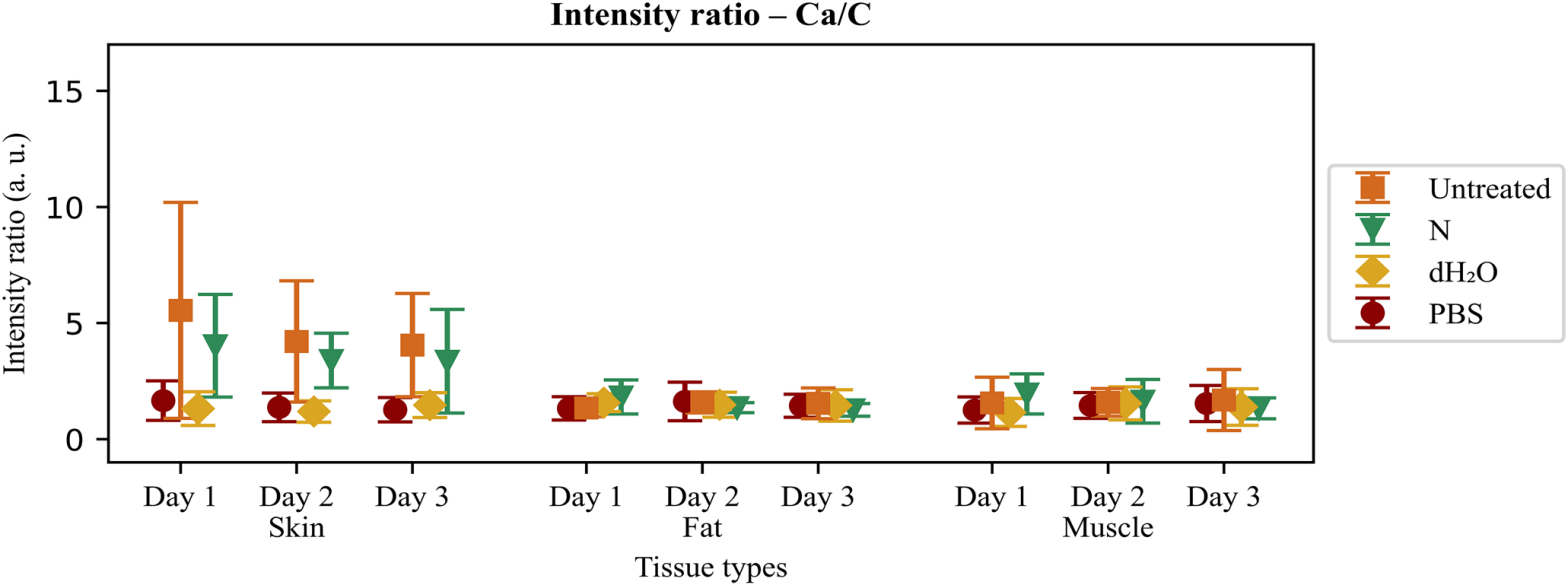
The intensity ratios of Ca/C for porcine skin, fat and muscle are shown for the different sample preparation methods.

The smallest differences can be seen in the Ca/C intensity ratio, as shown in Figure 6. Larger differences are only visible in porcine skin, where the untreated samples have the highest intensity ratios, which decrease slightly as with the liquid N samples. The embedded samples behave in the same way for all tissue types. Over the measurement period, there is no significant change in the intensity ratio; it remains almost constant. Like the liquid N samples, the untreated samples also behave consistently for fat and muscle. Li et al. [20] also analysed muscle, skin and fat from the porcine and found out that skin had the highest Ca intensities.

### 4.4 Ratio Mg/C

**FIGURE 7:**
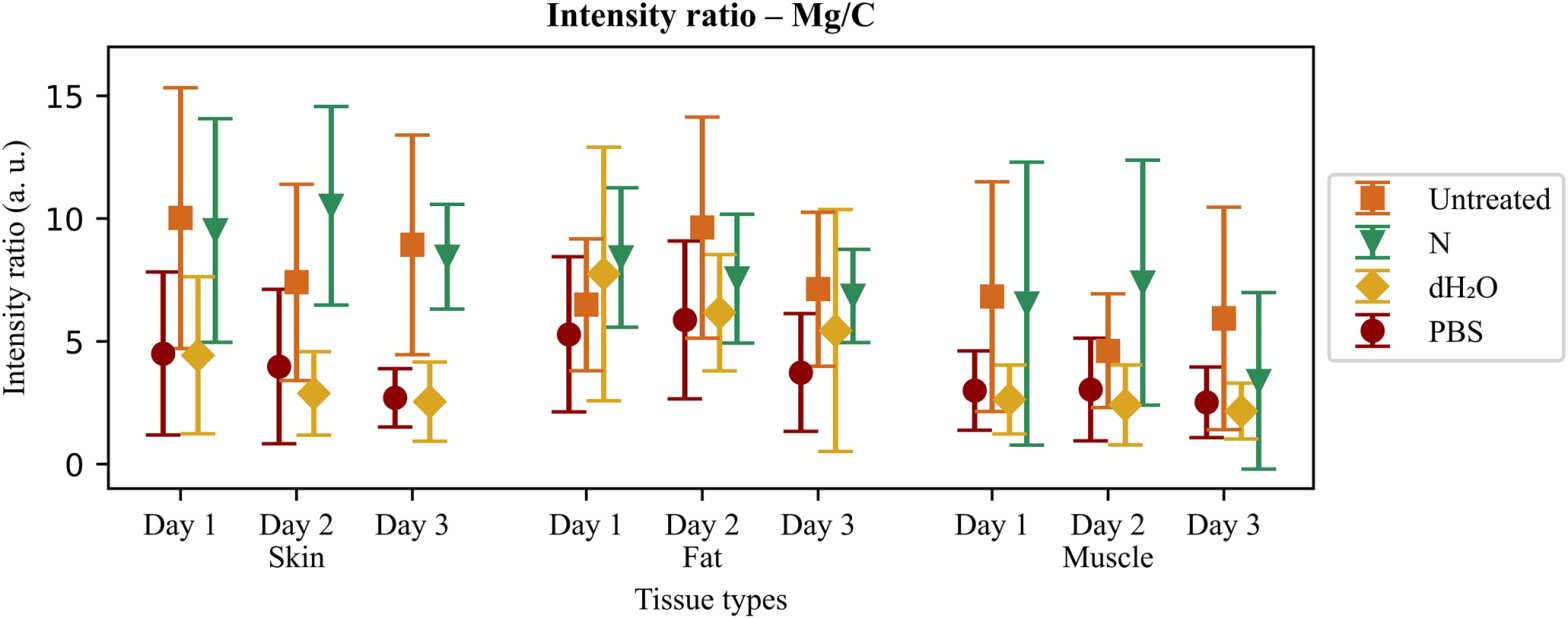
Intensity ratios of Mg/C. These are for porcine skin, fat and muscle. The ratios are for the different sample preparation methods.

The Mg/C intensity ratio exhibits greater variance in the standard distribution, see Figure 7. There are also greater differences between the sample preparation methods. High Mg intensities are observed in porcine skin for the liquid N and untreated samples, with both ratios decreasing only slightly on the last day of measurement. The immersed samples have significantly lower intensity ratios, which decrease further by the third day. For fat, the samples embedded in PBS have the lowest intensity ratio, while those cut with liquid N have the highest. The intensity ratio decreases for all sample preparation methods. Only the untreated samples show a slight increase on the third day. Significant differences between the untreated and liquid N samples are evident in the muscle. Here, the embedded samples have the lowest intensity ratios, which decrease over the measurement period. The liquid N samples show a slight increase in intensity ratios on Day 2, followed by a decrease on Day 3.

### 4.5 Nitrogen Samples

Higher intensities for frozen samples than for untreated samples, as reported in the literature [35], could not be clearly demonstrated in this study. While significantly higher intensities were evident for the Na/C ratio in porcine skin, as well as for Mg/C, this was not the case for the other tissue types. However, due to the significantly better optical spectra, it is assumed that the liquid N samples interact better with the laser pulse than the others. As demonstrated by Harun et al. [19], freezing the samples in liquid N improved the LIBS signal for Na/C and Mg/C. By contrast, it can be assumed that the liquid absorbed by the embedded samples hindered the laser–material interaction. While it is easier to cut the samples after placing them in liquid N, this method does not lead to better LIBS signals.

### 4.6 Embedded Samples

This study found that PBS and dH_2_O produced similar effects on the spectrum. The only differences observed were in the skin and the Mg/C intensity ratio between embedded and unembedded samples. In terms of sample handling, however, it was found that unembedded samples are easier to handle because embedded samples tend to splash during measurement due to the absorbed liquid. Teng et al. [52] analysed glioma, a kind of brain tumour, with LIBS using paraffin‐embedded samples and fresh samples. He found out that the fresh samples had lower intensities due to the wet surface [52]. The similar behaviour of immersed samples confirms the literature, which states that immersion causes stabilisation of the elements [41, 42]. The previously assumed chemical equilibrium between the elements in the liquid and tissue, and its effect on the spectrum, could not be confirmed when compared with samples immersed in dH_2_O. Embedding made the measurement itself more difficult, as the setup had to be cleaned frequently. There was no improvement in the LIBS signal.

### 4.7 Untreated Samples

The increase in intensity ratios for the untreated samples is consistent with previous observations. Jansen et al. [48], who investigated the Na content of rat organs shortly after death, also found that Na content increases after death. They attributed this to the diffusion of Na into the extracellular space after death [48]. Because Na is also present in PBS, it can be assumed that there was no matrix effect, as the results of this study are consistent with those in the literature. Nagaraj et al. [51] investigated biochemical and physicochemical changes in goat meat during post‐mortem ageing. They found that there is an increase in Ca^2+^ after death due to biochemical changes. Farmer et al. [53] investigated Mg changes in the human eye after death and also discovered that Mg concentration increases after death, depending on the post‐mortem interval. Figures 5, 6, 7 clearly show that Ca and Na are more reactive than Mg. This is evident from the smaller differences in intensity ratios between tissue types and measurement duration for Ca and Na than for Mg. This can be explained by the periodic table. Na is an alkali metal that requires only a small amount of energy for a chemical reaction [54]. Mg and Ca are alkaline earth metals, with Ca listed one row below Mg in the periodic table. This means that Ca requires less ionisation energy for a chemical reaction than Mg [54]. Due to the loose structure of fat and muscle compared to skin, a faster chemical reaction also occurs because the structure allows for faster diffusion. This is reflected in more constant intensity ratios.

## 5 Limitation

To counteract the influence of biological factors such as age, sex and diet on the porcine samples, samples from three animals were used. However, the butcher did not have more precise data on the animals, such as their origin, sex or time of slaughter. To counteract variance in the LIBS spectra, three samples of each tissue type were analysed at three different locations. Due to the flexible structure of the samples, it was not possible to cut them with a vibratome; therefore, the samples were cut with a scalpel, resulting in nonplanar surfaces. These nonplanar surfaces can cause the laser to be slightly out of focus, resulting in lower spectrum intensities. Due to the samples being cut by hand, different sizes were obtained for each tissue type. An attempt was made to keep the weight similar.

To avoid measuring the air, the laser was slightly focused into the sample surface. Since the concentrations of N, O and H in the tissues are much higher than in the atmosphere, the contribution of the atmosphere to the total N, O and H signals can be neglected. All the sample analyses could be assumed to be affected equally by the elements from the atmosphere, meaning this effect can be neglected.

## 6 Conclusion

In summary, this study uses CF‐LIBS analysis to demonstrate how sample preparation methods influence the LIBS spectra of porcine skin, fat and muscle. Embedding in PBS or dH_2_O has a minimal impact on spectral characteristics compared to untreated samples or samples cut with liquid N, with differences in intensity ratios being limited to specific elements such as Mg and Na in porcine skin. Spectral reproducibility and intensity remain consistent across preparation methods, confirming that preparation is unnecessary.

### 6.1 Future Scope

This study demonstrates that CF‐LIBS can be applied to in vivo measurements and shows that tissue can be analysed with CF‐LIBS under different preparation conditions. The next step is to compare these results with in vivo measurements on porcine tissue. Future studies should investigate how spectral features change in the period shortly after an animal dies. Such experiments would help to distinguish true in vivo signals from post‐mortem alterations, and would further validate CF‐LIBS for biological and potentially clinical applications.

## Author Contributions

**Edith Böhmer:** conceptualisation, investigation, methodology, software, writing – original draft. **Oana‐Maria Thoma:** conceptualisation, methodology. **Maximilian J. Waldner:** supervision. **Florian Klämpfl:** supervision, writing – review and editing. **Michael Schmidt:** supervision. **Martin Hohmann:** supervision.

## Funding

The authors gratefully acknowledge funding of the Erlangen Graduate School in Advanced Optical Technologies (SAOT) by the Bavarian State Ministry for Science and Art. The authors would like to thank the German Research Foundation (DFG‐Deutsche Forschungsgemeinschaft) for its support. This work was achieved in the context of the DFG‐project ‘Calibration‐free Laser‐Induced Breakdown Spectroscopy (LIBS) for the elemental analysis of tissue’ (502911968).

## Conflicts of Interest

The authors declare no conflicts of interest.

## Data Availability

Data will be made available on request.
